# Gut Microbiota Composition and Plasma Metabolomic Profile Are Associated with Amyloid Pathology and Cognitive Performance in Patients with Mild Cognitive Impairment

**DOI:** 10.3390/nu18132200

**Published:** 2026-07-07

**Authors:** Marina Mora-Ortiz, Magdalena P. Cardelo, Esther Porras-Pérez, Alejandro Serrán-Jiménez, Carlos A. Ledesma-Escobar, Feliciano Priego-Capote, Cristina Conde-Gavilán, Eduardo Agüera-Morales, Rafael Pineda Reyes, Maria M. Malagon, Elena M. Yubero-Serrano, Antonio Camargo, Niki Katsiki, José López-Miranda, Pablo Perez-Martinez

**Affiliations:** 1 Lipids and Atherosclerosis Unit, Department of Internal Medicine, Reina Sofia University Hospital, 14004 Cordoba, Spain; malenipc023@gmail.com (M.P.C.);; 2 Department of Medical and Surgical Sciences, Universidad de Cordoba, 14004 Cordoba, Spain; 3 GC-09, Nutrigenomics and Metabolic Syndrome, Maimonides Institute of Biomedical Research of Cordoba (IMIBIC), 14004 Cordoba, Spain; 4 Department of Cell Biology, Physiology and Immunology, Maimonides Institute of Biomedical Research of Cordoba (IMIBIC), University of Cordoba, 14004 Cordoba, Spain; 5 Department of Analytical Chemistry and Nanochemistry University Institute, Universidad de Cordoba, 14071 Cordoba, Spain; 6 CIBER de Fragilidad y Envejecimiento Saludable (CIBERFES), Instituto de Salud Carlos III, 28029 Madrid, Spain; 7 Neurology Service, Maimonides Institute for Biomedical Research in Cordoba (IMIBIC), Reina Sofia University Hospital, 14004 Cordoba, Spain; 8 CIBER Pathophysiology of Obesity and Nutrition (CIBEROBN), Carlos III Health Institute, 28029 Madrid, Spain; 9 Department of Food and Health, Fat Institute, Spanish National Research Council (CSIC), 41013 Seville, Spain; 10 Department of Nutritional Sciences and Dietetics, International Hellenic University, 57400 Thessaloniki, Greece; 11 School of Medicine, European University Cyprus, Nicosia 2404, Cyprus

**Keywords:** mild cognitive impairment, gut microbiota, 16S rRNA, untargeted metabolomics, amyloid-beta 42/40, ADAScog11, gut–brain axis, Mediterranean diet

## Abstract

Background/Objectives: The gut–brain axis and systemic metabolic dysregulation are increasingly implicated in Alzheimer’s disease (AD) pathogenesis. This study aimed to characterize gut microbiota and plasma metabolomic profiles associated with amyloid pathology and cognitive impairment in patients with mild cognitive impairment (MCI). Methods: A cross-sectional multi-omics baseline analysis was performed in 47 MCI patients enrolled in a randomized, double-blind, crossover dietary intervention trial (NCT05029765). Gut microbiota composition was assessed by 16S rRNA sequencing (*n* = 47), and plasma metabolomics by untargeted LC-MS/MS (*n* = 45 after exclusion of two PCA-defined metabolomic outliers). Patients were stratified according to plasma amyloid-beta 42/40 ratio (BA42/40) and ADAScog11 score, representing complementary biomarkers of amyloid burden and cognitive impairment, respectively. Results: Higher amyloid burden and worse cognitive performance were associated with significant gut microbiota alterations, including increased alpha diversity and distinct beta diversity profiles. Differential abundance analyses consistently showed enrichment of *Bacteroides*-associated taxa and *Akkermansia*, alongside depletion of short-chain fatty acid-producing genera such as *Faecalibacterium*, *Blautia*, and *Phascolarctobacterium*. Plasma metabolomics identified a coherent signature associated with elevated BA42/40, characterized by accumulation of secondary bile acid sulfates and depletion of sphingolipids, neuroactive steroids, and anti-inflammatory lipid mediators, including pregnenolone sulfate, resolvin E1, and anandamide. A valid OPLS-DA discriminant model was obtained for BA42/40, whereas no predictive model was achieved for ADAScog11. Critically, this dissociation, characterized by significant microbiota differences but no metabolomic separation for ADAScog11, is itself an informative finding, suggesting that gut microbiota dysbiosis and plasma metabolomic alterations are not equally coupled to both dimensions of MCI pathophysiology. Conclusions: MCI patients with greater amyloid pathology and cognitive impairment exhibited gut microbiota dysbiosis. However, metabolic associations were observed only for BA42/40, but not for ADAScog11. These findings provide a mechanistic framework for evaluating the impact of Mediterranean diet and probiotic interventions in the longitudinal phase of the trial.

## 1. Introduction

Mild cognitive impairment (MCI) represents a clinically critical transitional stage between normal aging and Alzheimer’s disease (AD), the most common cause of dementia [1]. Annual progression rates from MCI to AD range from 6.8% in community settings to 8.1% in clinical cohorts [2], and the absence of effective disease-modifying pharmacological treatments [3] has refocused research attention on lifestyle-based interventions, particularly dietary strategies, as modifiable factors capable of delaying or preventing cognitive deterioration [4,5].

Two interconnected biological processes have gained particular prominence. The gut–brain axis is the bidirectional communication network linking the intestinal microbiota and the central nervous system through neuroendocrine, immune, and metabolic routes, and it is increasingly implicated in neurodegeneration [6,7]. Reductions in butyrate-producing bacteria and increases in pro-inflammatory taxa have been associated with cognitive decline and Alzheimer’s pathology [8]. In parallel, metabolic dysregulation has emerged as another key feature of disease progression. Untargeted plasma metabolomics has identified perturbations in bile acid, lipid, and amino acid metabolism accompanying cognitive impairment, suggesting metabolic reprogramming that may contribute to neuroinflammation and neurodegeneration [9,10].

In this study, we report a cross-sectional multi-omics analysis of baseline samples from the trial (NCT05029765), a randomized, double-blind, Latin-square crossover dietary intervention study evaluating the effect of Mediterranean diet ± probiotic supplementation on cognitive function in 47 MCI patients [11]. We characterize gut microbiota and plasma metabolome in relation to two complementary biomarkers: the plasma amyloid-beta 42/40 ratio (BA42/40), which links with amyloid pathology burden [12], and the ADAScog11 score, a validated functional cognitive scale [13]. Plasma sampling was selected because it is less invasive than CSF collection or PET imaging and is therefore more compatible with repeated assessment in a dietary intervention trial. The parallel use of both biomarkers, which proved only partially concordant, allowed us to identify microbiota and metabolome signatures specific to different dimensions of the MCI phenotype.

## 2. Materials and Methods

### 2.1. Study Design and Patients

This cross-sectional analysis was embedded within the trial NCT05029765 [11], a randomized, double-blind, Latin-square crossover dietary intervention conducted at the Maimónides Biomedical Research Institute (IMIBIC) and Reina Sofía University Hospital, Córdoba, Spain. Ethical approval for the trial was granted by the Human Investigation Review Committee, Reina Sofía University Hospital (protocol 1496/27/03/2009), under a broader ongoing nutritional intervention programme. All participants provided written informed consent. The inclusion criteria considered an age ≥ 60 years; Clinical Dementia Rating = 0.5; MMSE > 23; RBANS delayed memory ≤ 85; Geriatric Depression Scale < 6. From 166 screened candidates, 47 completed baseline assessment and were included. Patients were dichotomously stratified using a median split of the plasma BA42/40 ratio (cohort median = 1.422); the 23 patients above the median were classified as High-BA42/40 and the 24 at or below the median as Low-BA42/40. An analogous median split was applied for ADAScog11 (21 High, 26 Low).

### 2.2. Stool Sample Collection and 16S rRNA Sequencing

Fecal samples were collected at baseline prior to any intervention, frozen at −80 °C within 30 min, and batch-processed. Total microbial DNA was extracted using bead-beating with chemical lysis. The V3–V4 hypervariable region of the 16S rRNA gene was amplified and sequenced on an Illumina MiSeq platform (2 × 300 bp paired-end). DADA2 was used for quality filtering, denoising, and chimera removal. ASVs were taxonomically assigned using SILVA v138 and relabeled sequentially (ASV_1 to ASV_N; mapping in Supplementary Table S1A,B).

### 2.3. Microbiome Bioinformatic Pipeline

All community-level analyses were performed in MicrobiomeAnalyst v2.0 [14]. The original table comprised 8539 ASVs across 47 samples (4,181,380 total reads; mean 88,965 reads/sample; range 32,245–170,101). After low-count filtering (≥4 counts in ≥20% of samples) and low-variance filtering (bottom 10% by IQR), 2231 ASVs were retained. Normalization implied total sum scaling (TSS) without rarefaction. Alpha diversity (Chao1) was compared via two-sample t-test with Benjamini–Hochberg (BH) correction. Beta diversity was assessed via PCoA of unweighted UniFrac distances, tested by PERMANOVA (999 permutations). Taxonomic differential abundance was assessed via heat tree (Wilcoxon rank-sum, *p* < 0.05), core microbiome analysis (≥0.01% in ≥20% of samples), and LEfSe (LDA ≥ 2.0). Non-assigned taxa were excluded from all tables and interpretations. For the heat tree visualization of the ADAScog11 comparison, the direction of comparison was inverted (Low vs. High) to align color coding with the BA42/40 panel, ensuring that yellow/warm tones consistently indicate taxa enriched in higher cognitive burden groups across both panels.

### 2.4. ASV-Level Differential Abundance—edgeR

Univariate differential abundance at the individual ASV level was assessed using a negative binomial exact test (equivalent to edgeR’s approach), implemented in MicrobiomeAnalyst’s ‘DE analysis’ module, applied directly to filtered count data with TMM normalization. Results are expressed as log_2_ fold-change (High/Low), logCPM, raw *p*-values, and BH FDR-adjusted q-values (threshold: FDR < 0.05). Detailed results were provided in Supplementary Table S2A–C and visualized as Manhattan plots by phylum.

### 2.5. Plasma Sample Collection and Untargeted Metabolomics

Fasting EDTA plasma was collected at baseline, centrifuged immediately, and stored at −80 °C until batch analysis. Untargeted LC-MS/MS metabolomic profiling was performed following the analytical platform established by our group for dietary intervention cohorts [15]. Briefly, metabolites were extracted by protein precipitation, separated on a reversed-phase C18 column, and detected using a Q-TOF instrument in positive and negative electrospray ionization modes. Features were annotated against in-house reference libraries. The same pipeline was applied to both the BA42/40 and ADAScog11 comparisons.

### 2.6. Metabolomics Statistical Pipeline

Raw feature matrices were processed in MetaboAnalyst v6.0 [16]. Missing values were imputed using the LoD method (1/5 of the minimum positive value per feature). Normalization included sum normalization, followed by log_10_ transformation and mean centering. A low-variance filter (IQR, bottom 10%) was applied, yielding 395 annotated features. Two samples were identified as outliers during PCA inspection of the metabolomics data (DC7043 and DC7036) and were removed from the metabolomics analysis only, yielding a working metabolomics dataset of 45 samples. These two patients were retained in all microbiome analyses (*n* = 47), as their gut microbiota profiles did not show any anomalies. The microbiome (*n* = 47) and metabolomics (*n* = 45) analyses therefore operate on overlapping but asymmetrical datasets; all multi-omics interpretations were made at the group level and did not require one-to-one patient matching.

The pipeline considered three statistical approaches which were applied to each comparison: (i) fold change (FC) analysis on pre-normalization data (threshold |FC| ≥ 2.0); (ii) two-sample Wilcoxon rank-sum test (*p* < 0.05 nominal; BH-FDR correction applied, no features survived); and (iii) OPLS-DA using the ropls R package [17] with Variable Importance in Projection (VIP) scores. Random Forest classification (500 trees) was run as a confirmatory machine learning approach.

Model quality criteria for OPLS-DA considered R^2^Y (explained variance in Y) and Q^2^ (cross-validated predictive ability). A model was considered valid and interpretable only if Q^2^ > 0 and no evidence of overfitting was observed; models with Q^2^ ≤ 0 were rejected as non-predictive. For models retained for interpretation, robustness was further assessed by label permutation testing. This rule was applied before biological interpretation. Thus, the BA42/40 OPLS-DA model, which fulfilled the Q^2^ acceptance criterion, was challenged by 100 random class-label permutations. In the MetaboAnalyst/ropls output, permutation-test *p*-values are calculated from cumulative model statistics across predictive and orthogonal components. The ADAScog11 OPLS-DA model was rejected because Q^2^ was negative and was therefore not subjected to validation for interpretive purposes.

## 3. Results

### 3.1. Cohort and Sequencing Summary

Forty-seven MCI patients were included (mean age 73.1 ± 6.2 years; 57.4% male; BMI 27.9 ± 3.4 kg/m^2^). Elevated systolic blood pressure (136.9 ± 14.4 mmHg), low-grade inflammation (hsCRP 8.8 ± 6.2 mg/dL), and moderate Mediterranean diet adherence (MEDAS 7.5 ± 2.0/14) were characteristic of the cohort. Energy intake was 2066.5 ± 506.3 kcal/day with 24.8 ± 11.0 g fibre/day (Table 1). Of the 47 patients, 23 were High-BA42/40 and 24 Low-BA42/40; 21 were High-ADAScog11 and 26 Low-ADAScog11. Cross-tabulation of both stratifications showed partial overlap between classification schemes (57.4% concordance), although the association was not statistically significant (χ^2^ = 0.516, *p* = 0.473). After filtering, 2231 ASVs were retained from the original 8539 for all microbiome analyses.

### 3.2. Alpha and Beta Diversity

**Figure 1:**
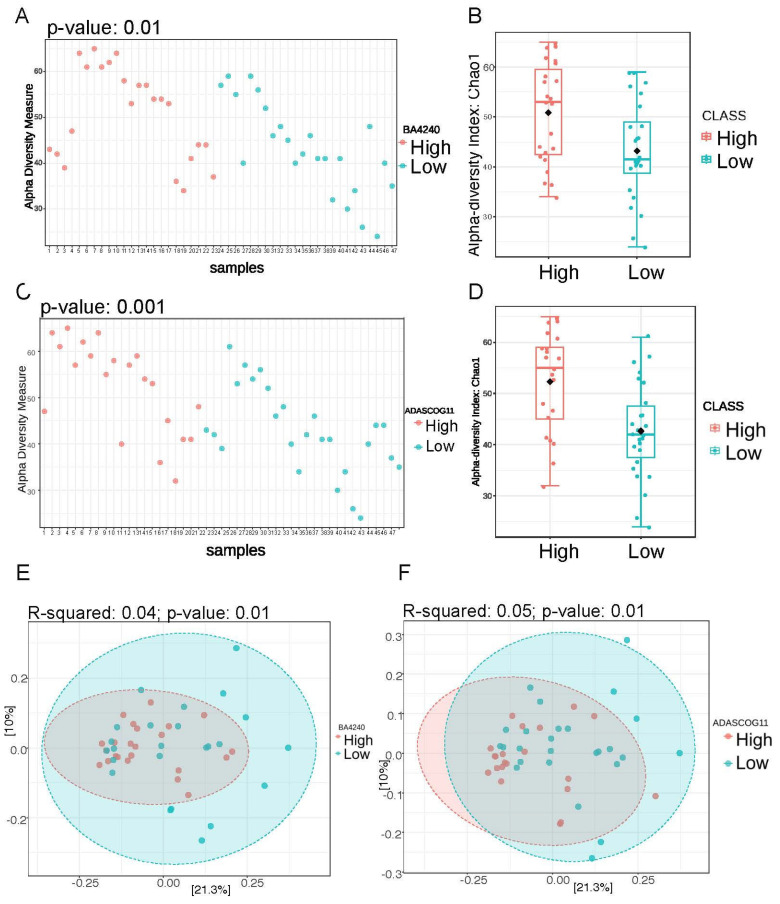
Alpha and beta diversity. (**A**,**C**) Chao1 richness per patient for BA42/40 and ADAScog11 stratifications; (**B**,**D**) boxplot comparisons (High vs. Low); (**E**,**F**) PCoA of unweighted UniFrac distances with 95% confidence ellipses. PERMANOVA with 999 permutations. R^2^: proportion of total compositional variance explained by group membership.

Chao1 richness was significantly higher in the High-BA42/40 group (mean 50.9 ± 10.0 vs. 43.2 ± 10.0; t = 2.622, *p* = 0.012) and in the High-ADAScog11 group (52.3 ± 9.9 vs. 42.7 ± 9.3; t = 3.405, *p* = 0.001; Table 2, Figure 1A–D). PCoA of unweighted UniFrac distances revealed visual separation between the High and Low groups for both biomarkers, confirmed by PERMANOVA: BA42/40 (F = 1.944, R^2^ = 0.041, *p* = 0.014) and ADAScog11 (F = 2.238, R^2^ = 0.047, *p* = 0.010; Table 3, Figure 1E,F). These effect sizes indicate that 4.1–4.7% of total phylogenetic compositional variance was attributable to cognitive group membership.

### 3.3. Heat Tree and Core Microbiome

**Figure 2:**
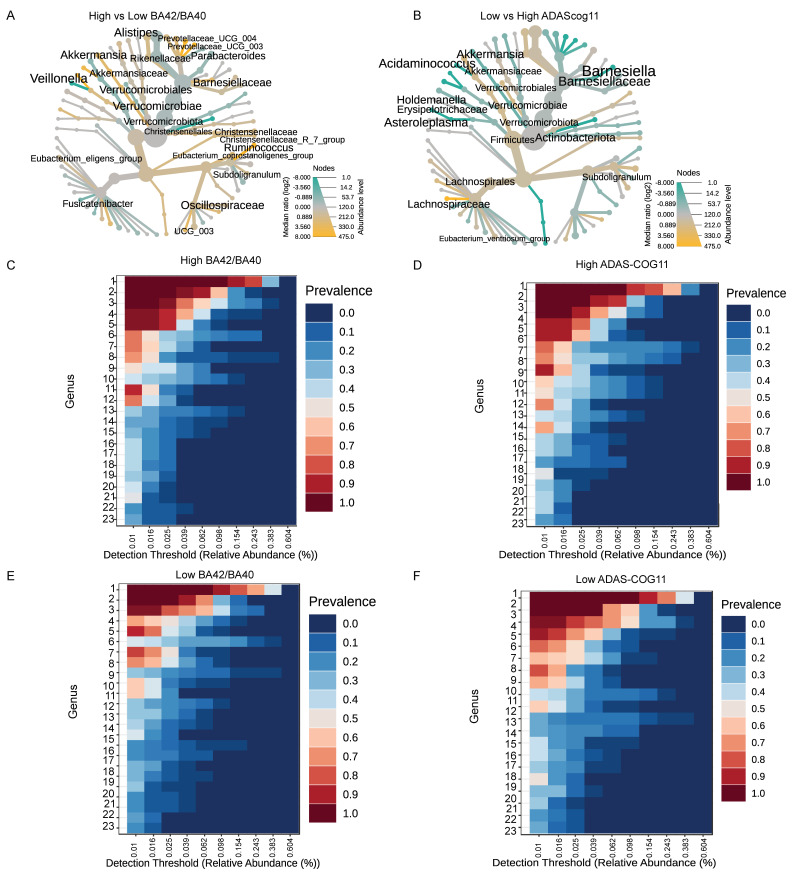
(**A**) Heat tree—High vs. Low BA42/40: node size represents abundance; color represents log_2_ median ratio (yellow = enriched in High; blue = enriched in Low; *p* < 0.05). (**B**) Heat tree—ADAScog11 comparison (Low vs. High orientation inverted for visualization consistency): node size represents abundance; color represents log_2_ median ratio, with the same color mapping as panel (**A**) (yellow = enriched in higher cognitive burden group; blue = enriched in lower cognitive burden group). This inversion was applied to ensure consistent cross-panel interpretation of taxonomic enrichment patterns. (**C**–**F**) Core microbiome heatmaps showing genus-level prevalence at increasing relative abundance thresholds for High-BA42/40 (**C**), High-ADAScog11 (**D**), Low-BA42/40 (**E**) and Low-ADAScog11 (**F**). Non-assigned features excluded. Genus in heatmaps are the next: Panel (**C**), from 1 to 23: *Bacteroides, Not_Assigned, Faecalibacterium, Alistipes, UCG_002, Akkermansia, Eubacterium_eligens_group, Prevotella_9, Phascolarctobacterium, Prevotellaceae_NK3B31_group, Parabacteroides, Subdoligranulum, Escherichia_Shigella, Ruminococcus, Barnesiella, Pseudobutyrivibrio, Blautia, Sutterella, Eubacterium_siraeum_group, UCG_005, Odoribacter, Streptococcus, Eubacterium_ruminantium_group*. Panel (**D**), from 1 to 23: *Bacteroides, Not_Assigned, Faecalibacteriu, UCG_002, Alistipes, Prevotella_9, Akkermansia, Parabacteroides, Phascolarctobactterium, Prevotellaceae_NK3B31_group, Sutterella, Barnesiella, Aciadaminococcus, odoribacter, Eubacterium_siraeum_group, Blautia, UCG_005, Pseudobutyrivibrio.* Panel (**E**), from 1 to 23: Bacteroides, Not_Assigned, Faecalibacteriu, Phascolarctobacterium, Alistipes, Prevotella_9, Parabacteroides, UCG_002, Escherichia_Shihgella, Blautia, Eubacterium_eligens_group, Prevotellaceae_NK3B31_group, Sutterella, Akkeremansia, Subdoligranulum, Dialister, Aciadaminococcus, Eubacteriium_siraeu_group, Pseudobutyrivibrio, Odoribacter, Paraprevotella, CAG_352, Fusicatenibacter, Ruminococcus_torques_group. Panel (**F**): *Bacteroides, Not_Assigned, Faecalibacterium, Alistipes, UCG_002, Phascolarctobacterium, Parabacteroides, Eubacterium_eligens_group, Prevotella_9, Blautia, Escherichia_Shigella, Prevotellaceae_NK3B31_group, Pseudobutyrivibrio, Akkermansia, Sutterella, Subdoligranulum, Eubacterium_siraeum_group, Odoribacter, UCG_005, Eubacterium_rumminantium_group*.

Heat tree analysis (BA42/40, Wilcoxon *p* < 0.05) showed that at the phylum level, Firmicutes was more abundant in the Low-BA42/40 group (log_2_ ratio = 0.39, *p* = 0.050), while Verrucomicrobiota (log_2_ = −2.75, *p* = 0.015) and Actinobacteriota (log_2_ = −0.62, *p* = 0.038) were enriched in the High group. At genus level, Lachnospiraceae members were more abundant in High-BA42/40 (*p* = 0.036), while Barnesiella (*p* = 0.002), *Asteroleplasma* (*p* = 0.001), and *Acidaminococcus* (*p* = 0.016) were depleted. For ADAScog11, an analogous taxonomic redistribution was observed (Figure 2A,B).

Core microbiome analysis showed *Bacteroides* universally present (100%) in all groups. *Akkermansia* prevalence was higher in High-ADAScog11 (71.4% vs. 42.3%; mean abundance 6.0% vs. 1.2%). *Prevotella*_*9* followed the same direction (76.2% vs. 42.3%). *Faecalibacterium* maintained near-universal prevalence but lower mean abundance in High-ADAScog11 (7.2% vs. 12.2%). *Blautia* prevalence was reduced in High-ADAScog11 (38.1% vs. 57.7%; Figure 2C–F).

### 3.4. LEfSe: Discriminant Genera

LEfSe (LDA ≥ 2.0, nominal *p* < 0.05, FDR < 0.1) was used as an exploratory taxonomic discriminant analysis and interpreted together with heat tree, core microbiome, and ASV-level edgeR results. The final BA42/40 and ADAScog11 LEfSe outputs identified several candidate discriminant genera at the nominal significance level. However, none of the detected taxa remained significant after correction for multiple testing. Consequently, no robust taxonomic discriminants were identified by LEfSe for either clinical variable. Given the exploratory nature of these findings, LEfSe results were not considered as standalone evidence of differential abundance and were interpreted only in the context of the complementary microbiome analyses described above. Full LEfSe outputs for the BA42/40 and ADAScog11 comparisons are provided in Supplementary Tables S3A and S3B, respectively.

### 3.5. ASV-Level Differential Abundance (edgeR)

**Figure 3:**
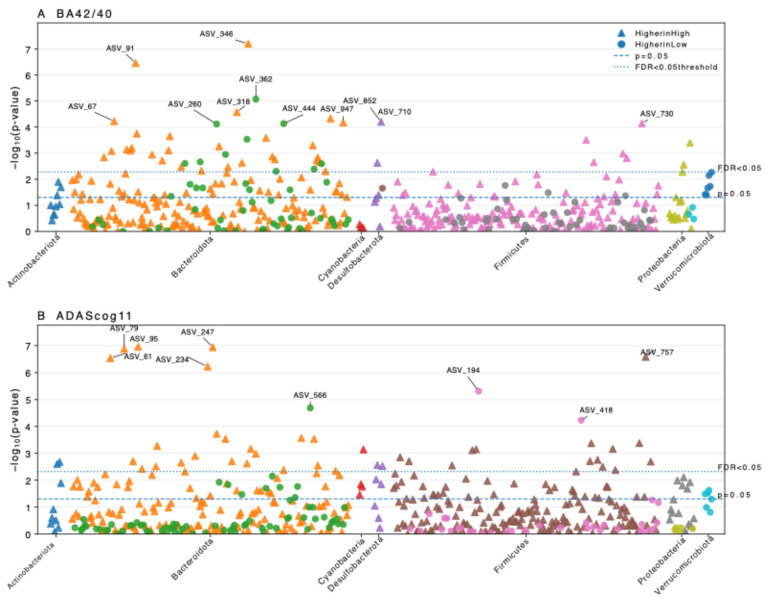
Manhattan plots of ASV-level differential abundance. Manhattan plots of ASV-level differential abundance based on edgeR negative binomial models for (**A**) BA42/40 and (**B**) ADAScog11. Each point represents one ASV. Point colour indicates phylum, and point shape indicates the direction of enrichment based on log_2_FC: triangles denote ASVs enriched in the High group, whereas circles denote ASVs enriched in the Low group. The Y-axis shows −log_10_(*p*-value). Labelled ASVs exceed −log_10_(*p*) > 4. Horizontal dashed lines indicate the nominal significance threshold, *p* = 0.05 [−log_10_(0.05) ≈ 1.30], whereas dotted lines indicate the panel-specific raw *p*-value threshold corresponding to BH-FDR < 0.05. Non-assigned ASVs were excluded.

Differential abundance analysis identified 50 significant ASVs for BA42/40 (FDR < 0.05): 40 enriched in High and 10 in Low (Figure 3A and Table 4). For ADAScog11, 46 ASVs were significant: 43 enriched in High and 3 in Low (Figure 3B and Table 4). In both comparisons, the majority of up-regulated ASVs belonged to Bacteroidota (genus *Bacteroides*), with log_2_FC values ranging from 1.9 to 5.8. For BA42/40, ASVs enriched in the Low group included *Prevotellaceae* UCG-004 (ASV_362, log_2_FC = −3.80, FDR = 1.4 × 10^−3^) and two unclassified Bacteroidota ASVs. For ADAScog11 (Table 5), the three ASVs enriched in the Low group were *Asteroleplasma* (ASV_194, log_2_FC = −3.28, FDR = 3.3 × 10^−4^), *Phascolarctobacterium* (ASV_418, log_2_FC = −2.93, FDR = 3.1 × 10^−3^), and an unclassified Bacteroidota (ASV_566, log_2_FC = −3.21, FDR = 1.2 × 10^−3^). Selected results are shown in Supplementary Materials Table S2A,B.

### 3.6. Plasma Metabolomics—BA42/40 (Valid OPLS-DA)

**Figure 4:**
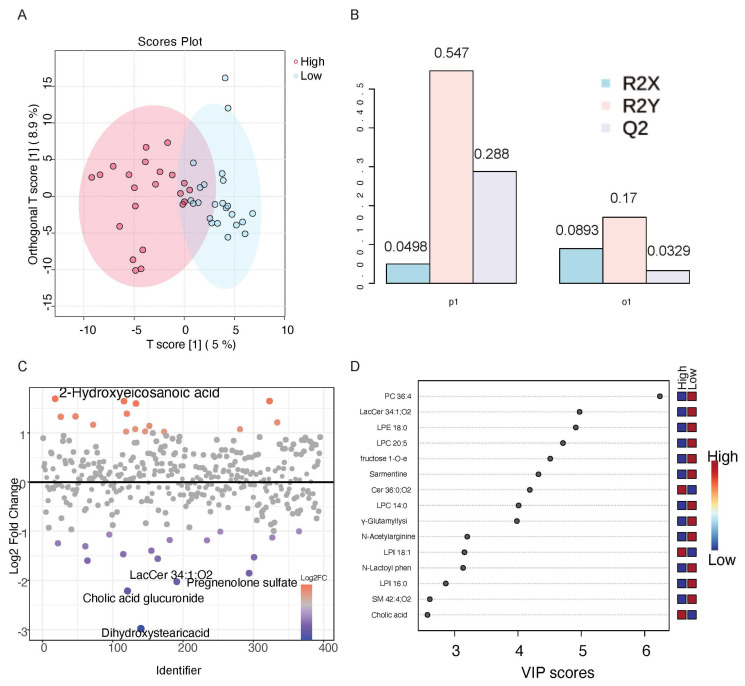
Untargeted plasma metabolomics—BA42/40 comparison (*n* = 45; OPLS-DA valid model). (**A**) OPLS-DA scores plot: predictive component T score [1] (5% of X variance) vs. orthogonal score o1 (8.9%). (**B**) Model quality parameters: R^2^X, R^2^Y, and Q^2^ by component. (**C**) Fold change scatter plot: log_2_FC (High/Low) vs. feature identifier; orange = enriched in High, blue = enriched in Low; labelled features exceed |FC| ≥ 2. (**D**) OPLS-DA VIP barplot (top 15 features); bar color indicates mean concentration higher in High (orange) or Low (blue) BA42/40. Note: labeled features in panel (**C**) were identified using a fold change threshold of |FC| ≥ 2.0 applied to pre-normalization group means (a magnitude filter, not a statistical test). Features shown in panel (**D**) were selected by OPLS-DA VIP score ≥ 1.0 in the predictive component, derived from the normalized data within the supervised model. The two criteria are complementary and selection sets may differ. Permutation testing was performed using 100 random class-label permutations. The retained model statistics reported in the Results correspond to the predictive component [R^2^Y(p1) = 0.547, Q^2^(p1) = 0.288], whereas the permutation-test panel reports cumulative statistics across predictive and orthogonal components [R^2^Y(cum) = 0.717, Q^2^(cum) = 0.321]. No permuted model exceeded the observed cumulative R^2^Y or Q^2^ values (empirical *p* < 0.01; 0/100 permutations).

OPLS-DA against BA42/40 (*n* = 45 after removal of two metabolomic outliers, DC7043 and DC7036) yielded a retained model with predictive-component statistics R^2^Y(p1) = 0.547 and Q^2^(p1) = 0.288 (R^2^X: 5.0% predictive, 8.9% orthogonal). See Figure 4A,B for futher details. A subsequent 100-label permutation test supported model robustness. The permutation module reported cumulative observed statistics across predictive and orthogonal components: R^2^Y(cum) = 0.717 and Q^2^(cum) = 0.321, corresponding to R^2^Y(p1 + o1) and Q^2^(p1 + o1), respectively. No permuted model exceeded the observed cumulative R^2^Y or Q^2^ values, yielding empirical *p* < 0.01 for both statistics (0/100 permutations). These results reduced concern that the retained BA42/40 OPLS-DA separation was driven by overfitting. Full model metrics and permutation results are provided in Supplementary Table S4A.

Fold change analysis identified 32 features with |FC| ≥ 2.0: 15 elevated in High-BA42/40 and 17 in Low-BA42/40 (Table 6, Figure 4C). Twenty-five features reached nominal Wilcoxon significance (*p* < 0.05); none survived BH-FDR correction, consistent with the cohort size. Full fold-change and Wilcoxon rank-sum outputs are provided in Supplementary Tables S5A and S5B, respectively.

Metabolites elevated in the High-BA42/40 group were predominantly secondary bile acid sulfates (chenodeoxycholic acid sulfate, deoxycholic acid 3-sulfate, cholesterol sulfate), primary bile acids (cholic acid), hydroxylated fatty acids (2-hydroxyeicosanoic acid), and bile pigments (biliverdin, stercobilin). Metabolites depleted in High-BA42/40 included the sphingolipid LacCer 34:1; O2, pregnenolone sulfate (a neuroactive steroid), resolvin E1 (omega-3 anti-inflammatory oxylipid), anandamide (endocannabinoid), C-glycosyltryptophan, and multiple lysophospholipids. OPLS-DA VIP analysis identified PC 36:4 (VIP C1 = 6.24), LacCer 34:1; O2 (VIP = 4.97), LPE 18:0 (VIP = 4.91), and LPC 20:5 (VIP = 4.71) as the top discriminant features (Table 7, Figure 4D).

The full VIP table is provided in Supplementary Table S4B, and the corresponding S-plot output in Supplementary Table S4C.

### 3.7. Plasma Metabolomics—ADAScog11 (Rejected OPLS-DA; Descriptive FC Only)

The OPLS-DA model for the ADAScog11 comparison was non-predictive (R^2^Y = 0.313, Q^2^ = −0.157 for the predictive component; Q^2^ = −0.339 for the orthogonal component) and was therefore rejected following pre-established criteria. This result was consistent with a two-sample t-test (no significant features) and Random Forest classification (OOB error = 44%, not better than chance). Accordingly, no supervised model-based interpretation was performed for the ADAScog11 metabolomic comparison. Full descriptive details of the rejected model can be found in the Supplementary Materials Table S6A–C. For descriptive purposes, fold change analysis identified 34 features with |FC| ≥ 2.0: 8 elevated in High-ADAScog11 and 26 elevated in Low-ADAScog11.. Metabolites elevated in the High group included sphingomyelins (SM 32:1; O2, SM 34:1; O2, SM 35:1; O2), 2-hydroxyeicosanoic acid, and C-glycosyltryptophan. Metabolites elevated in the Low group overlapped substantially with the BA42/40 pattern, including dihydroxystearic acid, LPI 18:1, LacCer 34:1; O2, glycocholic acid, cholic acid glucuronide, pregnenolone sulfate, taurine-conjugated bile acids, anandamide, and γ-glutamyllysine. These descriptive findings should be interpreted with caution given model rejection. Full descriptive details of the rejected ADAScog11 metabolomics model and associated descriptive outputs are provided in Supplementary Table S6A–C. These results are provided for transparency only and are not interpreted as evidence of a valid ADAScog11 metabolomic discriminant model.

## 4. Discussion

This study provides a baseline multi-omics characterization of 47 MCI patients using gut microbiota profiling (16S rRNA), ASV-level differential abundance analysis, and untargeted plasma metabolomics. Our findings reveal that MCI patients with greater amyloid pathology burden (High-BA42/40) and those with greater functional cognitive impairment (High-ADAScog11) exhibited convergent yet distinct microbiota signatures at baseline, while plasma metabolomic dysregulation was most clearly demonstrated in relation to the amyloid biomarker.

The observation of higher Chao1 richness in the higher-burden groups for both cognitive biomarkers is counterintuitive at first glance, as greater microbial diversity is generally associated with better health outcomes. However, this apparent paradox may reflect ecological restructuring rather than functional improvement. Increased richness can occur alongside the depletion of dominant functionally important taxa and therefore does not necessarily indicate a healthier microbial ecosystem [18]. In our cohort, the concurrent depletion of abundant taxa associated with gut homeostasis, including *Faecalibacterium* and *Blautia*, supports this interpretation rather than a true gain of beneficial diversity. Similar paradoxes have been reported in other disease contexts where diversity metrics dissociate from microbial functional capacity.

The enrichment of multiple *Bacteroides* ASVs in both high-burden groups, showed consistently across edgeR analysis, heat tree, core microbiome, and LEfSe, is the most robust taxonomic finding of this study. *Bacteroides* is a highly heterogeneous genus; while some species are considered commensals, others are associated with intestinal inflammation and increased intestinal permeability [19]. The concurrent depletion of *Faecalibacterium prausnitzii*—the single largest contributor to intestinal butyrate production—is mechanistically important. *Faecalibacterium* exerts potent anti-inflammatory effects via butyrate-mediated inhibition of NF-κB signaling, and its reduction has been associated with impaired intestinal barrier function and systemic endotoxemia, both of which are implicated in AD neuroinflammation [20]. Similarly, the depletion of *Phascolarctobacterium* (enriched in Low-ADAScog11), a genus that ferments succinate to produce propionate, suggests a broader reduction in SCFA-producing capacity in more cognitively impaired patients.

The enrichment of *Akkermansia muciniphila* in High-ADAScog11 patients warrants careful interpretation. While *Akkermansia* is generally regarded as a beneficial mucosal bacterium at low-to-moderate abundance, high abundance has been associated with mucosal degradation and increased intestinal permeability in inflammatory contexts [21]. Its enrichment in high-cognitive-burden patients may therefore link a pro-inflammatory mucus-erosive state rather than a protective role. This context-dependent interpretation is consistent with the concurrent depletion of butyrate producers, which normally maintain mucosal integrity.

The plasma metabolomic analysis for BA42/40 yielded a statistically supported OPLS-DA model (R^2^Y = 0.547, Q^2^ = 0.288), revealing two major patterns of dysregulation. First, the accumulation of bile acid sulfates (chenodeoxycholic, deoxycholic, and cholesterol sulfates) in High-BA42/40 patients may show impaired hepatic sulfation capacity or altered gut microbial bile acid biotransformation; these processes are partly dependent on the microbiota itself, and the results thereby connect the microbiome and metabolomics findings. Second, the depletion of pregnenolone sulfate (a neuroactive steroid with GABAergic properties) and anandamide (an endocannabinoid with neuroprotective and anti-inflammatory effects) in High-BA42/40 patients suggests impairment of neuroactive lipid signaling pathways in those with greater amyloid burden [22,23]. In addition, resolvin E1 depletion could point towards reduced omega-3-derived anti-inflammatory capacity. Finally, the sphingolipid LacCer 34:1; O2 was identified as a top VIP metabolite, and its reduction in High-BA42/40 is consistent with reports of altered ceramide and glycosphingolipid metabolism in AD [24].

The failure of the OPLS-DA model to discriminate ADAScog11 groups (Q^2^ = −0.157) despite clear microbiotic differences between the same groups is an important negative finding. This dissociation suggests that, at baseline, plasma metabolomic profiles may be more separable along the amyloid-related biological axis than along the functional cognitive performance axis.

The BA42/40 ratio was measured in plasma, which represents a less invasive alternative to CSF-based amyloid measurement. While plasma amyloid biomarkers have lower specificity than CSF for confirming Alzheimer’s pathology, they could be considered a meaningful biological approach and have been previously used in several large cohort studies [25]. The observed metabolomic differences in relation to this plasma biomarker are therefore biologically plausible, while acknowledging that CSF confirmation would strengthen biomarker specificity.

The present study has several strengths: a well-characterized MCI cohort within a registered clinical trial; joint analysis of two independent cognitive biomarkers capturing different dimensions of MCI; three convergent microbiota analytical approaches; and an explicit model rejection criterion for metabolomics. Key limitations include the cross-sectional design at baseline (which precludes causal inference), a modest sample size (*n* = 47 for microbiota, *n* = 45 for metabolomics), a dichotomization of BA42/40 performed using a median split complicating the direct comparability with studies using established CSF-based or PET-confirmed amyloid thresholds, ASV-level 16S resolution (genus-level at best) and the absence of FDR-significant metabolomic features (consistent with sample size). Importantly, several factors known to influence gut microbiota composition, including medication use (e.g., statins, antihypertensive and antidiabetic therapies), and bowel-related factors, were not formally incorporated as covariates in the present baseline cross-sectional analyses. Although these variables were systematically collected within the parent trial, the modest sample size limited the feasibility of multivariable adjustment without substantial risk of overfitting. Consequently, residual confounding cannot be excluded. However, the longitudinal phase of this randomized Latin-square crossover trial is less susceptible to confounding by stable participant characteristics, including chronic medication use, because each participant serves as their own control across intervention periods. In addition, the use of plasma BA42/40 rather than CSF-based amyloid biomarkers is also a limitation regarding pathological specificity. The present analysis provides a baseline reference against which longitudinal changes induced by Mediterranean diet and probiotic supplementation will be evaluated.

## 5. Conclusions

MCI patients with greater amyloid pathology burden (High-BA42/40) and greater functional cognitive impairment (High-ADAScog11) exhibited significant and convergent gut microbiota differences, including higher species richness, phylogenetically distinct communities, enrichment of *Bacteroides* and *Akkermansia*, and depletion of butyrate-producing taxa including *Faecalibacterium*, *Blautia*, and *Phascolarctobacterium*. In contrast, plasma metabolomic alterations were only robustly associated with amyloid pathology. A valid OPLS-DA model identified a coherent metabolic signature linked to elevated BA42/40, including accumulation of bile acid sulfates and depletion of neuroactive steroids, anti-inflammatory lipid mediators, and glycosphingolipids. Critically, despite significant microbiota differences across ADAScog11 groups, no valid metabolomic model was obtained for functional cognitive impairment. This dissociation is itself an important finding, suggesting that gut microbiota dysbiosis and plasma metabolomic alterations are not equally coupled to both dimensions of MCI pathophysiology, and that circulating metabolic profiles may be more closely related to amyloid pathology burden than to functional cognitive performance. These complementary microbiota–metabolome findings provide a mechanistic framework for evaluating the effects of Mediterranean dietary and probiotic interventions during the longitudinal phase of the trial.

## Figures and Tables

**Table 1:** Baseline clinical and demographic characteristics (*n* = 47).

Variable	Mean ± SD	*n*/%
Age (years)	73.1 ± 6.2	—
Sex (% male)	—	57.4%
BMI (kg/m^2^)	27.9 ± 3.4	—
Waist circumference (cm)	99.6 ± 11.0	—
Systolic BP (mmHg)	136.9 ± 14.4	—
Diastolic BP (mmHg)	73.6 ± 10.3	—
Fasting glucose (mg/dL)	99.7 ± 22.6	—
HbA1c (%)	5.8 ± 0.7	—
Total cholesterol (mg/dL)	180.0 ± 35.0	—
LDL-c (mg/dL)	80.7 ± 34.3	—
hsCRP (mg/dL)	8.8 ± 6.2	—
Homocysteine (µmol/L)	22.1 ± 12.3	—
Energy intake (kcal/day)	2066.5 ± 506.3	—
Fibre intake (g/day)	24.8 ± 11.0	—
MEDAS score	7.5 ± 2.0	—
BA42/40 High/Low	—	23/24
ADAScog11 High/Low	—	21/26
Donepezil use	—	25.5%
Statin use	—	55.3%

Data are mean ± SD or percentage. BP, blood pressure; HbA1c, glycated haemoglobin; hsCRP, high-sensitivity C-reactive protein; MEDAS, Mediterranean Diet Adherence Screener (0–14 points).

**Table 2:** Alpha diversity (Chao1) by cognitive biomarker stratification.

Biomarker	Group	*n*	Chao1 Mean	Chao1 SD	t	*p*-Value
BA42/40	High	23	50.9	10.0	2.622	0.012
	Low	24	43.2	10.0		
ADAScog11	High	21	52.3	9.9	3.405	0.001
	Low	26	42.7	9.3		

t: two-sample *t*-test statistic; *p*-value: BH-adjusted.

**Table 3:** Beta diversity (unweighted UniFrac, PERMANOVA).

Comparison	Distance	F-Value	R^2^	*p*-Value	p.adj
BA42/40 High vs. Low	Unw. UniFrac	1.944	0.041	0.014	0.014
ADAScog11 High vs. Low	Unw. UniFrac	2.238	0.047	0.010	0.013

**Table 4:** Top differentially abundant ASVs, BA42/40 (edgeR, FDR < 0.05, selected).

ASV	Phylum/Genus	log_2_FC	logCPM	p-Raw	FDR	Direction
ASV_346	Bacteroidota/*Bacteroides*	+5.28	10.79	6.3 × 10^−8^	3.0 × 10^−5^	↑ High
ASV_91	Bacteroidota/*Bacteroides*	+5.66	12.38	3.5 × 10^−7^	8.4 × 10^−5^	↑ High
ASV_710	Bacteroidota/*Butyricimonas*	+3.59	9.22	4.7 × 10^−5^	3.3 × 10^−3^	↑ High
ASV_318	Bacteroidota/*Bacteroides*	+4.02	10.49	2.7 × 10^−5^	3.2 × 10^−3^	↑ High
ASV_852	Desulfobacterota/unclassified	+3.57	9.37	6.3 × 10^−5^	3.3 × 10^−3^	↑ High
ASV_947	Bacteroidota/*Coprobacter*	+3.26	8.78	6.7 × 10^−5^	3.3 × 10^−3^	↑ High
ASV_362	Bacteroidota/*Prevotellaceae* UCG-004	−3.80	10.00	8.5 × 10^−6^	1.4 × 10^−3^	↓Low
ASV_444	Bacteroidota/*Bacteroides*	−3.88	10.30	7.3 × 10^−5^	3.3 × 10^−3^	↓ Low
ASV_260	Bacteroidota/unclassified	−3.46	10.10	7.6 × 10^−5^	3.3 × 10^−3^	↓ Low

log_2_FC: log_2_(High/Low). Direction: group with higher abundance. Non-assigned and ambiguous taxa excluded. FC: fold change.

**Table 5:** Top differentially abundant ASVs, ADAScog11 (edgeR, FDR < 0.05, selected).

ASV	Phylum/Genus	log_2_FC	logCPM	p-Raw	FDR	Direction
ASV_95	Bacteroidota/*Bacteroides*	+5.79	12.16	1.1 × 10^−7^	2.1 × 10^−5^	↑ High
ASV_247	Bacteroidota/*Bacteroides*	+5.66	11.08	1.1 × 10^−7^	2.1 × 10^−5^	↑ High
ASV_79	Bacteroidota/*Bacteroides*	+5.60	12.33	1.3 × 10^−7^	2.1 × 10^−5^	↑ High
ASV_61	Bacteroidota/*Bacteroides*	+5.36	12.48	2.9 × 10^−7^	2.7 × 10^−5^	↑ High
ASV_757	Firmicutes/unclassified	+4.58	8.95	2.6 × 10^−7^	2.7 × 10^−5^	↑ High
ASV_450	Firmicutes/*Dialister*	+3.49	10.44	4.2 × 10^−4^	1.3 × 10^−2^	↑ High
ASV_616	Actinobacteriota/*Bifidobacterium*	+2.67	9.44	2.1 × 10^−3^	3.2 × 10^−2^	↑ High
ASV_194	Firmicutes/*Asteroleplasma*	−3.28	8.61	4.8 × 10^−6^	3.3 × 10^−4^	↓ Low
ASV_418	Firmicutes/*Phascolarctobacterium*	−2.93	8.91	5.8 × 10^−5^	3.1 × 10^−3^	↓ Low
ASV_566	Bacteroidota/unclassified	−3.21	8.84	2.0 × 10^−5^	1.2 × 10^−3^	↓ Low

log_2_FC: log_2_(High/Low). Direction: group with higher abundance. Non-assigned and ambiguous taxa excluded. FC: fold change.

**Table 6:** Metabolites with |FC| ≥ 2.0, BA42/40 comparison (selected; *n* = 45).

Metabolite	FC (H/L)	log_2_FC	Class/Pathway
2-Hydroxyeicosanoic acid	3.24	+1.70	Hydroxylated fatty acid
Stercobilin	3.14	+1.65	Bile pigment
Chenodeoxycholic acid sulfate	3.13	+1.65	Bile acid sulfate
Deoxycholic acid 3-sulfate	3.03	+1.60	Bile acid sulfate
Cholic acid	2.63	+1.39	Primary bile acid
5α-Pregnan-3β,20α-diol 20-sulfate	2.53	+1.34	Steroid sulfate
Cholesterol sulfate	2.04	+1.03	Steroid sulfate
Biliverdin	2.24	+1.17	Bile pigment
(enriched in Low)			
Dihydroxystearic acid	0.127	−2.98	Hydroxylated fatty acid
Cholic acid glucuronide	0.215	−2.21	Bile acid conjugate
LacCer 34:1; O2	0.245	−2.03	Sphingolipid/glycosphingolipid
Pregnenolone sulfate	0.277	−1.85	Neuroactive steroid
Arabitol	0.330	−1.60	Sugar alcohol
Resolvin E1	0.347	−1.53	Omega-3 oxylipid (anti-inflammatory)
C-Glycosyltryptophan	0.362	−1.47	Indole/tryptophan metabolite
Anandamide (20:4)	0.405	−1.31	Endocannabinoid

FC (H/L): fold change calculated from group means on pre-normalization data. Positive log_2_FC = higher in High-BA42/40. Upper section: enriched in High; lower section: enriched in Low.

**Table 7:** Top OPLS-DA discriminant metabolites, BA42/40 (VIP scores; model: R^2^Y = 0.547, Q^2^ = 0.288).

Metabolite	VIP (C1)	VIP (o1)	Class
PC 36:4	6.24	1.07	Phosphatidylcholine
LacCer 34:1; O2	4.97	3.67	Sphingolipid
LPE 18:0	4.91	1.44	Lysophospholipid
LPC 20:5	4.71	2.00	Lysophospholipid
Fructose 1-O-ethyl-β-glucopyranoside	4.51	5.05	Glycoside
Sarmentine	4.32	5.09	Amide alkaloid
Cer 36:0; O2	4.19	0.65	Ceramide
LPC 14:0	4.01	0.57	Lysophospholipid
γ-Glutamyllysine	3.98	1.35	Dipeptide
N-Acetylarginine	3.20	4.44	N-acetyl amino acid
N-Lactoyl phenylalanine	3.13	4.47	Amino acid conjugate
N-Acetyltyrosine	2.48	5.23	N-acetyl amino acid

VIP C1/o1: Variable importance in projection for predictive and orthogonal components. LPE, lysophosphatidylethanolamine; LPC, lysophosphatidylcholine; LacCer, lactosylceramide; PC, phosphatidylcholine.

## Data Availability

The datasets generated during the current study are available from the corresponding author upon reasonable request.

## Supplementary Materials

The following supporting information can be downloaded at: https://www.mdpi.com/article/10.3390/nu18132200/s1, Supplementary_Tables_organized.xlsx, including Table S1A: ASV sequence-to-label mapping; Table S1B: taxonomy table; Table S2A: full edgeR differential ASV results for BA42/40; Table S2B: full edgeR differential ASV results for ADAScog11; Table S2C: combined edgeR ASV results; Table S3A: LEfSe output for BA42/40; Table S3B: LEfSe output for ADAScog11; Table S4A: BA42/40 OPLS-DA model metrics and 100-permutation summary; Table S4B: full BA42/40 OPLS-DA VIP table; Table S4C: BA42/40 OPLS-DA S-plot output; Table S5A: BA42/40 fold-change output; Table S5B: BA42/40 Wilcoxon rank-sum output; Table S6A: ADAScog11 fold-change output, descriptive only; Table S6B: rejected ADAScog11 OPLS-DA model metrics; Table S6C: ADAScog11 VIP output from the rejected model, provided for completeness only and not interpreted.

## Abbreviations

The following abbreviations are used in this manuscript:

16S rRNA	16S ribosomal RNA gene
AD	Alzheimer’s disease
ADAScog11	Alzheimer’s Disease Assessment Scale–Cognitive Subscale 11
ASV	Amplicon Sequence Variant
BA42/40	Plasma amyloid-beta 42/40 ratio
BH	Benjamini-Hochberg correction
BMI	Body mass index
BP	Blood pressure
CIBEROBN	CIBER Pathophysiology of Obesity and Nutrition
CIBERFES	CIBER Frailty and Healthy Aging
CSF	Cerebrospinal fluid
DADA2	Divisive Amplicon Denoising Algorithm 2
FDR	False discovery rate
FC	Fold change
GABAergic	Gamma-aminobutyric acid-mediated
HbA1c	Glycated haemoglobin
hsCRP	High-sensitivity C-reactive protein
IQR	Interquartile range
LC-MS/MS	Liquid chromatography-tandem mass spectrometry
LDA	Linear discriminant analysis
LEfSe	Linear discriminant analysis effect size
LacCer	Lactosylceramide
LPC	Lysophosphatidylcholine
LPE	Lysophosphatidylethanolamine
LoD	Limit of detection
MCI	Mild cognitive impairment
MD	Mediterranean diet
MEDAS	Mediterranean Diet Adherence Screener
MMSE	Mini-Mental State Examination
NF-κB	Nuclear factor kappa B
OOB	Out-of-bag (error)
OPLS-DA	Orthogonal projections to latent structures discriminant analysis
PC	Phosphatidylcholine
PCoA	Principal coordinates analysis
PERMANOVA	Permutational multivariate analysis of variance
RBANS	Repeatable Battery for the Assessment of Neuropsychological Status
SCFA	Short-chain fatty acid
SILVA	SILVA ribosomal RNA sequence database
SM	Sphingomyelin
TMM	Trimmed mean of M-values
TSS	Total sum scaling
Ii ha	Variable importance in projection
